# The Simultaneous Electrochemical Detection of Catechol and Hydroquinone with [Cu(Sal-β-Ala)(3,5-DMPz)_2_]/SWCNTs/GCE

**DOI:** 10.3390/s141222274

**Published:** 2014-11-25

**Authors:** Lina Abdullah Alshahrani, Xi Li, Hui Luo, Linlin Yang, Mengmeng Wang, Songling Yan, Peng Liu, Yuqin Yang, Quanhua Li

**Affiliations:** Department of Chemistry, School of Chemistry, Chemical Engineering and Life Science, Wuhan University of Technology, Wuhan 430070, China; E-Mails: lamy7667@hotmail.com (L.A.A.); luohuiwhut@126.com (H.L.); chemyanglinlin@whut.edu.cn (L.Y.); doublemeng_1988@126.com (M.W.); yansongling@whut.edu.cn (S.Y.); chemliup@whut.edu.cn (P.L.); whyang2003@whut.edu.cn (Y.Y.); liquanhua@whut.edu.cn (Q.L.)

**Keywords:** copper(II) Schiff base complex, single-walled carbon nanotubes, modified electrode, catechol, hydroquinone, electrochemical detection

## Abstract

A glassy carbon electrode was modified with a copper(II) complex [Cu(Sal-β-Ala) (3,5-DMPz)_2_] (Sal = salicylaldehyde, β-Ala = β-alanine, 3,5-DMPz = 3,5-dimethylpyrazole) and single-walled carbon nanotubes (SWCNTs). The modified electrode was used to detect catechol (CT) and hydroquinone (HQ) and exhibited good electrocatalytic activities toward the oxidation of CT and HQ. The peak currents were linear with the CT and HQ concentrations over the range of 5–215 μmol·L^−1^ and 5–370 μmol·L^−1^ with corresponding detection limits of 3.5 μmol·L^−1^ and 1.46 μmol·L^−1^ (*S*/*N* = 3) respectively. Moreover, the modified electrode exhibited good sensitivity, stability and reproducibility for the determination of CT and HQ, indicating the promising applications of the modified electrode in real sample analysis.

## 1. Introduction

Catechol (CT) and hydroquinone (HQ), two dihydroxybenzene isomers, are widely used in industrial applications such as cosmetics, pesticides, flavoring agents, antioxidant, dyes and pharmaceutics [1–4]. They are highly toxic to both the environment and humans, even at very low concentrations. Moreover, they are difficult to degrade under typical ecological conditions. The high toxicity and low degradability has made CT and HQ important contaminants, which are considered as environmental pollutants by the US Environmental Protection Agency (EPA) and the European Union (EU) [5]. Therefore, it is very important to develop simple and rapid analytical methods for the determination of CT and HQ.

At present, the main determination methods are chromatography [6,7], spectrophotometry [8,9] and electrochemical methods [10,11]. Among all these techniques, spectrophotometry suffers from easy interference by related compounds and needs complicated pre-separation steps, which are unsuitable for multicomponent analysis. Chromatography has potential for the simultaneous detection of CT and HQ. However, it also requires time-consuming sample pretreatment steps and expensive instruments. Due to their merits of simple operation, prompt analysis and no need for complicated sample pretreatment, electrochemical methods have been developed as one of the most efficient alternatives in environmental analysis. However, the directly simultaneous determination of CT and HQ at conventional electrodes such as glassy carbon electrodes is difficult because the similar chemical structures lead to the overlap of their redox peaks. In order to overcome this problem, many materials such as nanomaterials [12,13], transition metal compounds [14,15] and conducting polymers [16,17], are widely applied to modify the electrodes. Carbon nanotubes are one of the most extensively used kinds of modifiers due to their unique nanostructure and extraordinary properties, including large surface area, excellent electrocatalytic activity and high stability [18,19]. Bu *et al.*, employed a glassy carbon electrode modified with multiwalled carbon nanotubes (MWNTs) and ionic liquids (IL) to simultaneously determinate HQ and CT [20]. The modified electrode showed two well-defined redox waves for HQ and CT in both cyclic voltammetry (CV) and differential pulse voltammetry (DPV). The experimental results with the enhancement of the redox peak current and the decrease of the peak-to-peak separation proved the excellent the electrocatalytic behaviors of the modified electrode. Meanwhile, transition metal complexes are also frequently employed as electrochemical mediators to modify the electrodes because of their catalysis at low potential, the reversible properties of the electrode reactiond and peculiar biological function [21,22]. Wu *et al.* developed a novel HQ sensor by electrochemically depositing a film of copper hexacyanoferrate (CuHCF) on a silicon electrode coated by a platinum layer [23]. The sensor showed good photocurrent responses to the addition of different concentrations of HQ with good stability, which provides a facile way of detecting HQ and avoids the use of an inconvenient reference electrode. The linear range for the detection of HQ was 1.0 × 10^−5^ to 2.0 × 10^−4^ mol·L^−1^, with a detection limit (*S*/*N* = 3) of 2.2 × 10^−6^ mol·L^−1^.

Among these complexes, Schiff base complexes have received much attention as electrocatalysts for the development of novel sensors because of their excellent electrocatalytic properties in the detection of many important analytes [24,25]. In this paper, a copper(II) complex with the Schiff base ligands salicylaldehyde and β-alanine [Cu(Sal-β-Ala)(3,5-DMPz)_2_] (Sal = salicylaldehyde, β-Ala = β-alanine, 3,5-DMPz = 3,5- dimethylpyrazole) was used to modified the electrode by electropolymerization. As carbon nanotubes have high accessible surface area and excellent electrocatalytic activity, single-walled carbon nanotubes (SWCNTs) were applied to immobilize copper(II) complex onto planar electrode surfaces, which help to ensure an extremely large surface and promote the electron-transfer reaction. The electrode modified with [Cu(Sal-β-Ala)(3,5-DMPz)_2_] and SWCNTs showed good electrocatalytic activities in the oxidation of CT and HQ. The simultaneous determination of CT and HQ was investigated at the modified electrode by DPV.

## 2. Experimental Section

### 2.1. Reagents and Apparatus

Single-walled carbon nanotubes were obtained from Shenzhen Nanotech Port Co., Ltd., (Shenzhen, China). The SWCNTs suspension (0.1 mg·mL^−1^) was prepared as described previously [26]. In brief, SWCNTs were treated using a mixture of concentrated H_2_SO_4_ and HNO_3_ solution for 3 h, and then filtered and washed with water until the pH was 7.0. The prepared SWCNTs (10 mg) were ultrasonically dispersed in 100 mL H_2_O to give a 0.1 mg·mL^−1^ black suspension. Catechol, hydroquinone and other chemicals purchased from Sinopharm Chemical Reagent Beijing Co., Ltd. (Beijing, China) were analytical reagent grade and used without further purification. [Cu(Sal-β-Ala) (3,5-DMPz)_2_] was prepared and purified according to the procedures reported previously [27]. Phosphate buffer solution (PBS, 0.1 mol·L^−1^) was prepared by mixing the stock solution of 0.1 mol·L^−1^ NaH_2_PO_4_ and 0.1 mol·L^−1^ Na_2_HPO_4_, and the pH was adjusted by NaOH or HCl. All the solutions were prepared with double distilled water.

### 2.2. The Preparation of the Modified Glassy Carbon Electrode

Before the modification, the glassy carbon electrode (GCE) was polished to a mirror-like surface with 1.0 μm and 0.3 μm Al_2_O_3_ paste, respectively. Then the electrode was cleaned ultrasonically with acetone and double distilled water in sequence. Finally the glassy carbon electrode was electrochemically cleaned in a 0.5 mol·L^−1^ sulfuric acid solution by continuous cycling from −0.35 to 1.5 V until a stable cyclic voltammogram was obtained.

The above SWCNTs suspension (6 μL, 0.1 mg·mL^−1^) was dropped onto the GCE surface, and exposed to the air for drying at 40 °C. This process could be repeated many times to fabricate SWCNTs-modified glass carbon electrodes (SWCNTs/GCEs).

Cu(II) complex-modified SWCNTs/GCE ([Cu(Sal-β-Ala)(3,5-DMPz)_2_]/MWCNTs/GCE) was prepared by cycling the potential between −0.8–1.2 V at 100 mV·s^−1^ in a dimethyl sulfoxide solution containing Cu(II) complex and 0.1 mol·L^−1^ NaNO_3_.

### 2.3. Electrochemical Measurements

All electrochemical experiments were carried out with a model CHI 660D electrochemical workstation (Shanghai Chenhua Equipments, Shanghai, China). A three-electrode cell was used, including a saturated calomel electrode (SCE) as the reference electrode, a platinum wire as the counter electrode and a bare or modified GCE (3 mm in diameter) as the working electrode. CV was employed to investigate the electrochemical behavior of CT and HQ at the modified electrode. DPV was applied for the single and simultaneous determination of CT and HQ, respectively.

## 3. Results and Discussion

### 3.1. Electrochemical Behaviors of CT and HQ at the Modified Electrode

**Figure 1.**
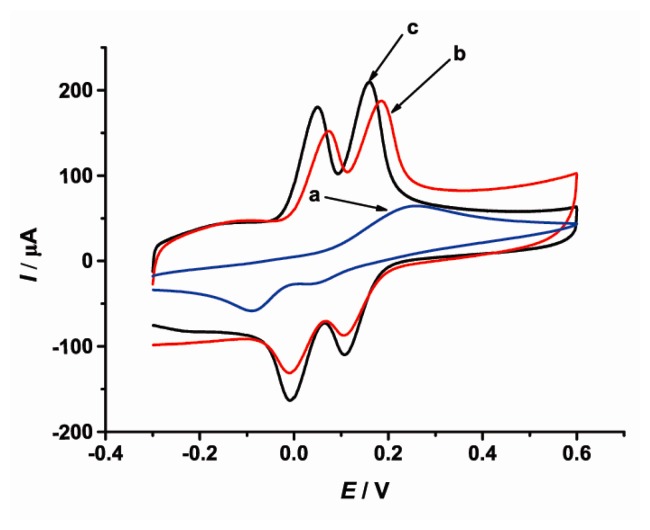
Cyclic voltammograms of 1.0 mmol·L−1 CT and 1.0 mmol·L−1 HQ in 0.1 mol·L−1 PBS (pH = 6.0) at (a) bare GCE; (b) SWCNTs/GCE; (c) [Cu(Sal-β-Ala) (3,5-DMPz)2]/SWCNTs/GCE. Scan rate: 100 mV·s−1.

The CVs of CT and HQ at bare GCE (Curve a), SWCNTs/GCE (Curve b) and [Cu(Sal-β-Ala) (3,5-DMPz)_2_]/SWCNTs/GCE (Curve c) are shown in Figure 1. At the bare GCE, the oxidation peaks of CT and HQ overlapped to form a wide peak at about 0.3 V, indicating that it was not possible to determine CT and HQ simultaneously. At the SWCNTs/GCE, well-defined oxidations of CT and HQ appeared at 0.07 V and 0.19 V, respectively. The oxidation peak potential difference between CT and HQ was 0.11 V. After [Cu(Sal-β-Ala)(3,5-DMPz)_2_] was electrodeposited on the SWCNTs-modified electrode, although the oxidation peak potential difference between CT and HQ changed little, the oxidation peak potential shifted negatively, showing the reversibility of the electrode reaction was enhanced. Meanwhile the oxidation peak currents of CT and HQ all increased. It could be concluded that CT and HQ were separated completely at [Cu(Sal-β-Ala)(3,5-DMPz)_2_]/SWCNTs/CCE and the simultaneous determination of the two species was feasible.

**Figure 2.**
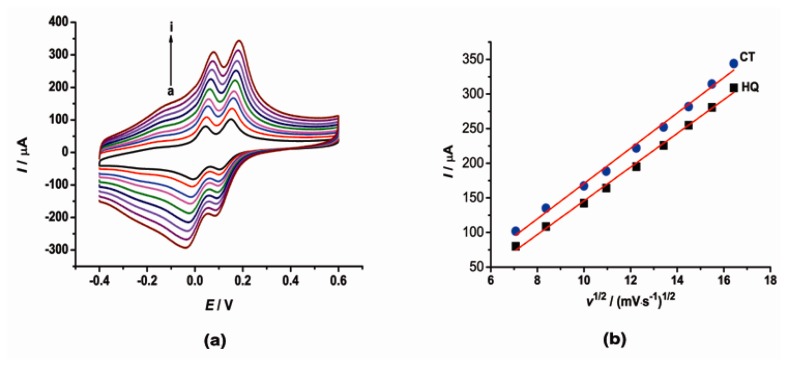
(**a**) Cyclic voltammograms of the [Cu(Sal-β-Ala)(3,5-DMPz)_2_]/SWCNTs/GCE in PBS solution (pH = 6.0) containing CT and HQ at a scan rate of 50, 70, 100, 120, 150, 180, 210, 240, 270 mV·s^−1^ (from a to i); (**b**) The relationship between oxidation peak currents and the square root of the scan rate.

The effect of scan rate on the cyclic voltammetric behaviors of CT and HQ at the [Cu(Sal-β-Ala) (3,5-DMPz)_2_]/SWCNTs/GCE was also investigated. The results are shown in Figure 2. The separations of the anodic and cathodic peaks, Δ*E*_p_, increased a little as the scan rate changed from 50 to 270 mV·s^−1^. In the meantime, the anodic peak currents of CT and HQ were proportional to the square root of the scan rate, which indicated that the oxidation of CT and HQ at the [Cu(Sal-β-Ala) (3,5-DMPz)_2_]/SWCNTs/GCE was a diffusion-controlled process.

### 3.2. The Optimization of Detecting Conditions

**Figure 3.**
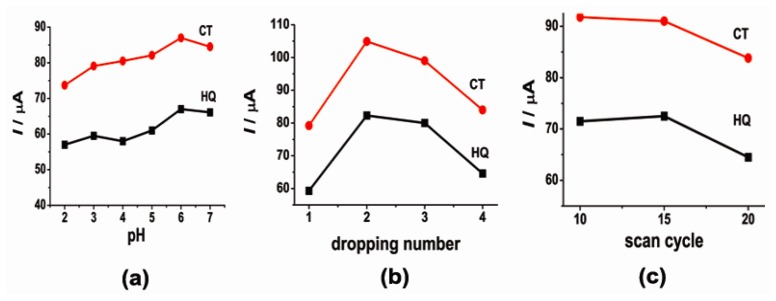
Dependence of the anodic peak current of CT and HQ in PBS solution (pH = 6.0) at [Cu(Sal-β-Ala)(3,5-DMPz)_2_]/SWCNTs modified electrode on (**a**) solution pH; (**b**) the amount of SWCNTs; (**c**) the cycle number of polymerization.

Because of the participation of protons in the electrochemical reaction, the solution pH is considered to affect the electrochemical behavior of CT and HQ. The effect of the solution pH on the electrochemical behaviors of CT and HQ was investigated in the pH range 2–7 by CV. The corresponding results are shown in Figure 3a. Apparently, the anodic peak currents of CT and HQ were greatly influenced by the solution pH. From pH 2 to 6, the anodic peak current increased as the pH increased. When pH equaled 6, the anodic peak currents increased up to 87.9 μA for CT and 67.4 μA for HQ. And when pH was higher than 6, the anodic peak currents decreased. Therefore, a value of 6 was chosen to determine CT and HQ in this work.

The peak currents of CT and HQ were also affected by the amount of SWCNTs on the electrode surface, which could be controlled by dropping the SWCNTs suspension many times. The relationship between the oxidation peak current of CT or HQ and dropping numbers is shown in Figure 3b. When the dropping numbers increased from 1 to 2, the anodic peak current of CT or HQ increased, but when the dropping numbers changed from 2 to 3, the anodic peak current decreased. Thus dropping twice was chosen for subsequent experiments.

Similarly, the thickness of the copper(II) complex film influenced the electrochemical behavior of CT and HQ at the [Cu(Sal-β-Ala)(3,5-DMPz)_2_]/SWCNTs/GCE. The thickness of the copper(II) complex film could be adjusted by changing the number of scan cycles during the electrodeposition of [Cu(Sal-β-Ala)(3,5-DMPz)_2_] on the electrode. According to Figure 3c, the most suitable number of scan cycles was 15.

### 3.3. The Individual Determination of CT and HQ at the Modified Electrode

**Figure 4.**
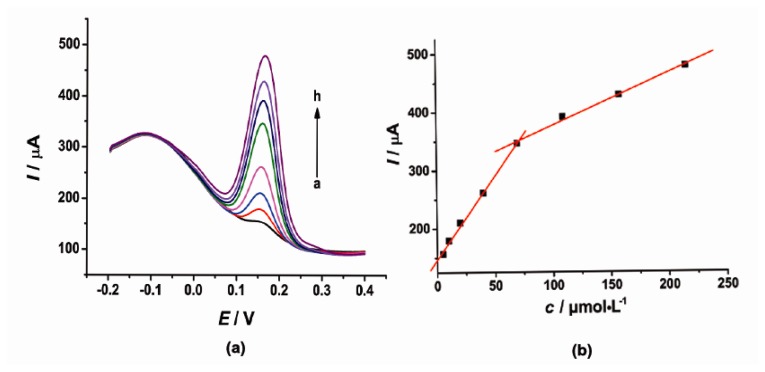
(**a**) DPV of CT in pH 6.0 PBS (0.1 mol·L^−1^ KCl) at the modified electrode, CT concentration range from 5 to 215 μmol·L^−1^ (a → h); (**b**) The corresponding curve of current response I *vs*. CT concentration.

**Figure 5.**
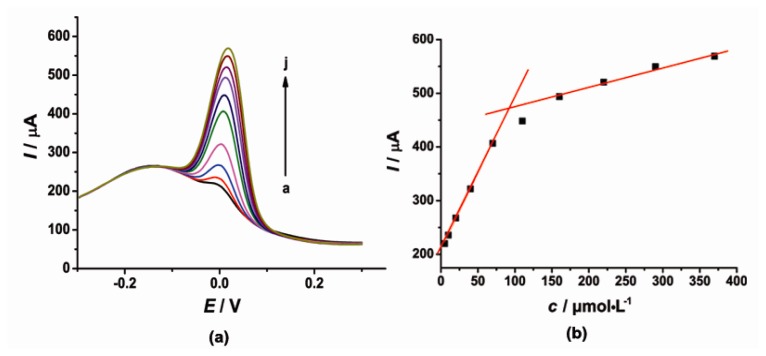
(**a**) DPV of HQ in pH 6.0 PBS (0.1 mol·L^−1^ KCl) at the modified electrode, HQ concentration range from 5 to 370 μmol·L^−1^ (a → j); (**b**) The corresponding curve of current response I *vs*. HQ concentration.

Under the optimal conditions, the individual determination of CT or HQ was investigated by DPV as shown in Figures 4 and 5. At the concentration range of 5–69.5 and 69.5–215 μmol·L^−1^, the current response signal (*I*) was proportional to the concentration (*c*) of CT. The linear regression equations were as follows: *I* = 2.864*c* + 148.5 (μA, *R*^2^ = 0.9985) and *I* = 0.8895*c* + 289.1 (μA, *R*^2^ = 0.9971). On the other hand, the oxidation peak current (*I*) of HQ was proportional to the concentration (*c*) with the linear regression equation of *I* = 2.853*c* + 207.7 (μA, *R*^2^ = 0.9998) in the concentration range of 5–70 μmol·L^−1^ and *I* = 0.3608*c* + 439.6 (μA, *R*^2^ = 0.9909) in the concentration range of 160–370 μmol·L^−1^. The detection limit for CT and HQ is 3.5 μmol·L^−1^ and 1.46 μmol·L^−1^ (*S*/*N* = 3), respectively.

### 3.4. The Simultaneous Determination of CT and HQ at the Modified Electrode

**Figure 6.**
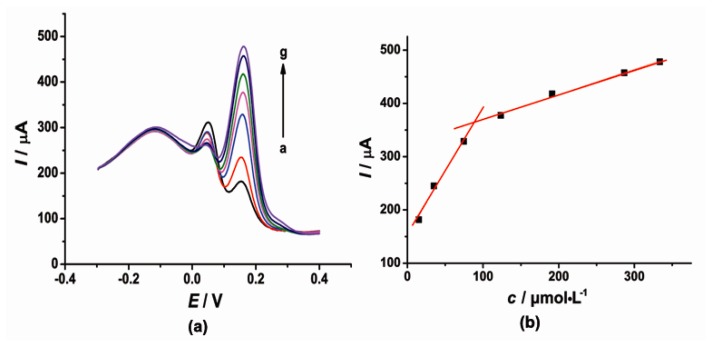
(**a**) DPV of 50 μmol·L^−1^ HQ and CT with concentrations ranging from 15 to 345 μmol·L^−1^ (a → g) in pH 6.0 PBS (0.1 mol·L^−1^ KCl) at the modified electrode; (**b**) The corresponding curve of current response I *vs*. CT concentration.

**Figure 7.**
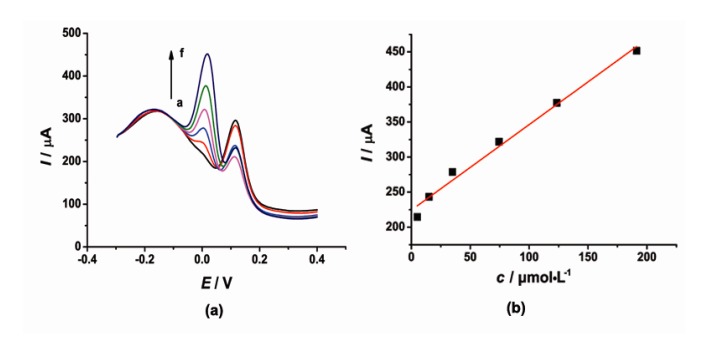
(**a**) DPV of 50 μmol·L^−1^ CT and HQ with concentrations ranging from 5 to 191 μmol·L^−1^ (a → f) in pH 6.0 PBS (0.1 mol·L^−1^ KCl) at the modified electrode; (**b**) The corresponding curve of current response I *vs*. HQ concentration.

DPV was also used for simultaneous determination of CT and HQ at the [Cu(Sal-β-Ala) (3,5-DMPz)_2_]/SWCNTs/GCE due to its high sensitivity. The determination of CT or HQ in their mixtures was investigated when the concentration of one species changed, whereas the other species remained constant. Figures 6 and 7 show the typical DPV curves of CT and HQ using the modified electrode with the increasing CT and HQ concentrations.

DPV curves (Figure 6) showed that as the concentration of CT increased, the current signal gradually increased. During the CT concentration range 15–75 and 125–345 μmol·L^−1^, the concentration and the current response of CT exhibited a good linear relationship respectively. The linear regression equations were as follows, *I*_pc_ = 2.421*c* + 151.7 (μA, *R*^2^ = 0.9939) and *I*_pc_ = 0.4699*c* + 322.9 (μA, *R*^2^ = 0.9966). Similarly, the current signal gradually increased with the increased concentration of HQ. The current was proportional to the concentration of HQ in the range of 5–191 μmol·L^−1^. The linear regression equation was as follows, *I*_pc_ = 1.219*c* + 224.3 (μA, *R*^2^ = 0.9939).

### 3.5. Reproducibility, Stability and Interference Study

The reproducibility of the [Cu(Sal-β-Ala)(3,5-DMPz)_2_]/SWCNTs/GCE was evaluated by detecting CT and HQ with five modified electrodes prepared using the same fabrication procedure. The relative standard deviation (RSD) is 2.45% for CT and 2.14% for HQ, which indicated the good reproducibility of the fabricated electrodes.

The stability of the sensing interface is also an important issue for the practical implementation of electrochemical detection. Therefore, the stability of the [Cu(Sal-β-Ala)(3,5-DMPz)_2_]/SWCNTs/GCE was investigated over a 2-week period. The modified electrode was stored dry at room temperature for two weeks and still retained about 96.6% of its original response for CT and 95.1% for HQ, revealing that the as-prepared electrode had excellent stability.

The major interferences in the simultaneous determination of CT and HQ (50 μmol·L^−1^) come from the coexisting substances, which may lead to an overlapping with the existing peaks. Some conceivable inorganic ions and organic compounds were tested at the modified electrode. It was found that 100-fold Mg^2+^, 100-fold Cu^2+^, 100-fold Fe^3+^, 100-fold K^+^, 100-fold Na^+^, 100-fold NO_3_^−^, 100-fold Cl^−^, 100-fold SO_4_^2−^, and 50-fold glucose, 50-fold ethanol, 50-fold cysteine, 50-fold lysine, 50-fold naphthol and 10-fold resorcinol had no obvious influences on the signals of CT and HQ and the tolerance limit was estimated to be less than 5% of the relative error, demonstrating an excellent tolerance to interference of the modified electrodes.

## 4. Conclusions

This work demonstrated the application of a copper(II) Schiff base complex and SWCNTs-modified electrode for the determination of CT and HQ. This fabricated electrochemical sensor showed an electrocatalytic activity towards the electrochemical response of CT and HQ with the enhancement of the oxidation peak currents of CT and HQ and the negative shift of the oxidation peak potentials. This modified electrode exhibited a wide linear range and low detection limit for the determination of HQ and CT. In addition, the results indicated that the modified electrode had good selectivity, high reproducibility and excellent stability in these electrochemical determinations. The excellent properties of the modified electrodes make them promising for application to real sample analysis.

## References

[1] Blackley R.L., Henry D.D., Smith C.J. (2001). Lack of correlation between cigarette mainstream smoke particulate phase radicals and hydroquinone yield. Food Chem. Toxicol..

[2] Ivanov D.P., Sobolev V.I., Pirutko L.V., Panov G.I. (2002). New Way of Hydroquinone and Catechol Synthesis Using Nitrous Oxide as Oxidant. Adv. Synth. Catal..

[3] Wang J., Park J.N., Wei X.Y., Lee C.W. (2003). Room-temperature heterogeneous hydroxylation of phenol with hydrogen peroxideover Fe^2+^, Co^2+^ ion-exchanged Naβ zeolite. Chem. Commun..

[4] Khachatryan L., Adounkpe J., Maskos Z., Dellinger B. (2006). Formation of Cyclopentadienyl Radical from the Gas-Phase Pyrolysis of Hydroquinone, Catechol, and Phenol. Environ. Sci. Technol..

[5] Suresh S., Vimal C.S., Indra M.M. (2012). Adsorption of catechol, resorcinol, hydroquinone, and their derivatives: A review. Int. J. Energy Environ. Eng..

[6] Gao W.H., Legido-Quigley C. (2011). Fast and sensitive high performance liquid chro-matography analysis of cosmetic creams for hydroquinone, phenol and sixpreservatives. J. Chromatogr. A.

[7] Penner A.N., Nesterenko N.P. (2000). Simultaneous determination of dihydroxybenzenes, aminophenols and phenylene diamines in hair dyes by high-performance liquid chromatography on hypercross-linked polystyrene. Analyst.

[8] Kai X.M., Shen Y.H., Zhang G.X., Xie A.J. (2005). Determination of pyrocatechol, resorcinol and hydroquinone by LM-BP neural network. Spectrosc. Spect. Anal..

[9] Afkhami A., Khatami H. (2001). Indirect kinetic-spectrophotometric determination ofresorcinol, catechol, and hydroquinone. J. Anal. Chem..

[10] Guo Q., Huang J., Chen P., Liu Y., Hou H., You T. (2012). Simultaneous determination of catechol and hydroquinone using electrospun carbon nanofibers modified electrode. Sens. Actuators B Chem..

[11] Chen K., Zhang Z., Liang Y., Liu W. (2013). A Graphene-Based Electrochemical Sensor for Rapid Determination of Phenols in Water. Sensors.

[12] Du H., Ye J., Zhang J., Huang X., Yu C. (2011). A voltammetric sensor based on graphene-modified electrode for simultaneous determination of catechol and hydroquinone. J. Electroanal. Chem..

[13] Huo Z., Zhou Y., Liu Q., He X., Liang Y., Xu M. (2011). Sensitive simultaneous determination of catechol and hydroquinone using a gold electrode modified with carbon nanofibers and gold nanoparticles. Microchim. Acta.

[14] Gan T., Sun J., Huang K., Song L., Li Y. (2013). A graphene oxide–mesoporous MnO_2_ nanocomposite modified glassy carbon electrode as a novel and efficient voltammetric sensor for simultaneous determination of hydroquinone and catechol. Sens. Actuators B Chem..

[15] Hua X., Fang P., Wang X., Chen Q. (2014). Transition-metal-catalyzed oxidation of metallic Sn in NiO/SnO_2_ nanocomposite. Chemistry.

[16] He K., Wang X., Meng X., Zheng H., Suye S. (2014). Amperometric determination of hydroquinone and catechol on gold electrode modified by direct electrodeposition of poly(3,4-ethylenedioxythiophene). Sens. Actuators B Chem..

[17] Saleh Ahammad A.J., Rahman M.M., Xu G., Kim S., Lee J. (2011). Highly sensitive and simultaneous determination of hydroquinone and catechol at poly(thionine) modified glassy carbon electrode. Electrochim. Acta.

[18] Song D., Xia J., Zhang F. (2015). Multiwall carbon nanotubes-poly (diallydimethylammonium chloride)-graphene hybrid composite film for simultaneous determination of catechol and hydroquinone. Sens. Actuators B Chem..

[19] Li D., Li Y., Songm W., Long Y. (2010). Simultaneous determination of dihydroxybenzene isomers using disposable screen-printed electrode modified by multiwalled carbon nanotubes and gold nanoparticles. Anal. Methods.

[20] Bu C., Liu X., Zhang Y., Li L., Zhou X., Lu X. (2011). A sensor based on the carbon nanotubes-ionic liquid composite for simultaneous determination of hydroquinone and catechol. Colloids Surf. B Biointerfaces.

[21] Fang B., Feng Y.H., Wang G.F., Zhang C.H., Gu A.X., Liu M. (2011). A uric acid sensor based on electrodeposition of nickel hexacyanoferrate nanoparticles on an electrode modified with multi-walled carbon nanotubes. Microchim. Acta.

[22] John Jeevagan A., Abraham John S. (2012). Electrochemical sensor for guanine using a self-assembled monolayer of 1,8,15,22-tetraaminophthalocyanatonickel(II) on glassy carbon electrode. Anal. Biochem..

[23] Wu H., Hu J., Li H., Li H. (2013). A novel photo-electrochemical sensor for determination of hydroquinone based on copper hexacyanoferrate and platinum films modified n-silicon electrode. Sens. Actuators B Chem..

[24] Dadamos T.R.L., Teixeir M.F.S. (2009). Electrochemical sensor for sulfite determination based on a nanostructured copper-salen film modified electrode. Electrochim. Acta.

[25] Zhang Z., Li X., Wang C., Zhang C., Liu P., Fang T., Xiong Y., Xu W. (2012). A novel dinuclear Schiff-base copper(II) complex modified electrode for ascorbic acid catalytic oxidation and determination. Dalton Trans..

[26] Liu X., Li X., Xiong Y., Huang Q., Li X., Dong Y., Liu P., Zhang C. (2013). A glassy carbon electrode modified with the nickel(II)-bis (1,10-phenanthroline) complex and multi-walled carbon nanotubes, and its use as a sensor for ascorbic acid. Microchim. Acta.

[27] Cao Y., Li X., Dong Y., Feng W., Xiong Y., Li X., Liu X. (2013). Electrocatalytic Properties of Schiff Base Copper(II) Complex Modified Electrode for Ascorbic Acid. J. Wuhan Univ. Technol..

